# Abstracts of the More Important Papers Read before the British Medical Association

**Published:** 1875-10

**Authors:** James Johnson

**Affiliations:** Birmingham


ABSTRACTS OF THE MORE IMPORTANT PAPERS READ BEFORE THE BRITISH MEDICAL ASSOCIATION.—*Rheumatic Fever and its Treatment.* By James Johnson, M. B., Birmingham.—The author brought forward for consideration, with regard to the causation of rheumatic fever, the non-conversion of starchy food into glucose, in consequence of irritation from improper food, or from exposure to cold and damp, and the conversion in the cæcum of the undigested starch into lactic acid, which, being absorbed, produced the phenomena of the disease. In regard to the treatment to be observed, he advocated the use of bicarbonate of soda given in enema, instead of potash. In twenty cases so treated, the temperature had gone down to below the normal state in the course of seven days.

*Remarks on Hooping-Cough and its Treatment with Carbolic Acid Vapor.* By Robert S. Lee, M. D., London.—Dr. Lee directed attention to the very slight variation in the annual mortality from hooping-cough, to its widespread geographical distribution, and to the results of various kinds of treatment. The conclusions which he deduced were the results of observation of six hundred cases, and the most important remarks were connected with the rise and fall of the disease at the first or second quarter of the year, and the frequency with which the disease was not diagnosed on account of the absence of the laryngeal spasm or whoop. The use of carbolic acid was recommended, as proving more satisfactory than any other kind of remedy ; and the method of administering it in the form of vapor, by means of the steam-draft inhaler, was explained. A solution of one part of carbolic acid in ten of water was kept as a standard for mixture, in the proportion of two drachms to four ounces of water. This was introduced into the inhaler, and every four hours its vapor was inspired for ten minutes or a quarter of an hour.

*On the Prevention and Management of Miscarriages.* By Arthur W. Edis, M. D., London.—A brief allusion was made to the mortality occasioned by miscarriages, the life of the fœtus being invariably sacrificed and the mother's life often jeopardized ; and not only this, but the fecundity of the female was often destroyed, from the effects of uterine disorder following a miscarriage, to say nothing of the distress and suffering often occasioned. In a series of two thousand cases observed by the author, there were no fewer than eleven hundred and forty-seven miscarriages compared with four thousand five hundred and eighty-eight children born at full time. Miscarriages were far too lightly esteemed, both by the public at large as well as practitioners. Patients with well marked flexion of the uterus, constitutional syphilis, chronic metritis, and numberless other affections, were allowed to go on aborting without sufficient pains being taken to prevent a recurrence. The prevention of miscarriages depended entirely upon proper measures being employed to avoid the contingency of a recurrence of the cause or combination of causes that induced the expulsion of the ovum in the first instance. To say that a patient had acquired the "habit of aborting" was merely asserting our ignorance of the cause, and expressing in other terms the fact that the woman aborted because she aborted. As regards the management of miscarriages, the necessity of a vaginal examination was strongly insisted on. The influence of ergot in the early stage, in arresting threatened miscarriages, was favorably alluded to, and the employment of carbolic acid injections where any portion of the products of conception were retained was urgently recommended. The author concluded by urging a more careful study of the subject; miscarriages being frequently the starting-point of a long course of uterine distress —dysmenorrhœa, sterility, etc.

*On Dysmenorrhœa.* By Mrs. E. Garrett Anderson, M. D.— In this paper, Mrs. Garrett Anderson discussed the following questions: 1. How far is the mechanical theory of dysmenorrhœa supported by facts? 2. What is the relation between mechanical or obstructive dysmenorrhœa and the so-called neuralgic, congestive, and rheumatic forms of the complaint? 3. To what extent ought the mechanical theory, if we accept it, to guide our treatment? With regard to the first question, Mrs. Garrett Anderson agreed with Dr. Marion Sims and Dr. Barnes, that the essential cause of dysmenorrhœa was retention of the uterine secretion. This view was supported by the curative influence of parturition. The author differed, however, from Dr. Sims when he denied the existence of constitutional dysmenorrhœa; for in a large number of cases the retention might depend on a constitutional condition. The anæmic, congestive, and rheumatic forms of dysmenorrhœa were commented on; also that dependent on uterine flexion. Mrs. Garrett Anderson did not believe in neuralgic dysmenorrhœa, as the term was commonly understood. The form thus described might depend on obstruction, or on abrasion of the os, with endometritis of the cervix or fundus.

Cases of ovarian origin were believed not to be common in early woman, not to be often primary. "Intermenstrual" dysmenorrhœa was not dysmenorrhœa at all, and was probably due to ovarian congestion. In regard to the treatment, Mrs. Garrett Anderson pointed out that there were facts which seem to indicate that, in accepting the mechanical theory of dysmenorrhœa, it is not necessary to adopt in the first instance, and in most cases, a mechanical line of treatment. Various constitutional conditions frequently gave rise to obstructive dysmenorrhœa, which could often be removed by constitutional measures.

*Neuralgic Dysmenorrhœa.* By Charles R. Drysdale, M. D.—The author thought that a salutary revolution was now setting in against the surgical doctrines held by Dr. Marion Sims and others upon dysmenorrhœa and its causes. Dr. Drysdale very rarely, indeed, witnessed any case where he had found any service to arise from operations on the uterus; whilst he had seen some cases of pelvic abscess and pelvic peritonitis occur from such interference. He was lately consulted by a patient, single, aged thirty-two, who had suffered since the age of sixteen from dysmenorrhœa, and who, on consulting two eminent specialists, was advised by the one to have recourse to incision of the cervix, and by the other to wear a pessary. In this case, the uterine sound passed in its normal direction without difficulty, and the patient had no leucorrhœa nor prolapse of the organ, which was quite normal in size. There was no ulceration of the os uteri, although another eminent specialist had considered this as the cause of her dysmenorrhœa. The author held that there was still too great a tendency to expect to find an evident physical cause for all painful menstruation. Spasm and neuralgia were quite sufficient to account for the vast majority of cases. Membranous shreds, also, were frequent causes of obstruction to the monthly flow. An illustrative case was recorded. The rational treatment of dysmenorrhœa commencing at an early period, consisted not in the use of pessaries, or of incision of the uterus, surely; but in the use of cold baths in the morning, with short walks in the open air afterwards; in hot baths, a few days previously to the menstrual periods; and in palliative treatment of the paroxysms by means of antispasmodics at the epoch of pain. Marriage sometimes cured such cases at once; at other times, it was of no use.—*Medical and Surgical Reporter.*

